# Development of 101 Gene-based Single Nucleotide Polymorphism Markers in Sea Cucumber, *Apostichopus japonicus*

**DOI:** 10.3390/ijms13067080

**Published:** 2012-06-08

**Authors:** Huixia Du, Zhenmin Bao, Jingjing Yan, Meilin Tian, Xiaoyu Mu, Shi Wang, Wei Lu

**Affiliations:** Key Laboratory of Marine Genetics and Breeding, College of Marine Life Science, Ocean University of China, Qingdao 266003, China; E-Mails: nevergiveupxia@sina.com (H.D.); zmbao@ouc.edu.cn (Z.B.); jing-yan12345@163.com (J.Y.); air880426@163.com (M.T.); muxiaoyu422@yahoo.cn (X.M.); swang@ouc.edu.cn (S.W.)

**Keywords:** single nucleotide polymorphism (SNP), *Apostichopus japonicus*, high resolution melting (HRM) analysis, marker-assisted selection (MAS)

## Abstract

Single nucleotide polymorphisms (SNPs) are currently the marker of choice in a variety of genetic studies. Using the high resolution melting (HRM) genotyping approach, 101 gene-based SNP markers were developed for *Apostichopus japonicus*, a sea cucumber species with economic significance for the aquaculture industry in East Asian countries. HRM analysis revealed that all the loci showed polymorphisms when evaluated using 40 *A. japonicus* individuals collected from a natural population. The minor allele frequency ranged from 0.035 to 0.489. The observed and expected heterozygosities ranged from 0.050 to 0.833 and 0.073 to 0.907, respectively. Thirteen loci were found to depart significantly from Hardy–Weinberg equilibrium (HWE) after Bonferroni corrections. Significant linkage disequilibrium (LD) was detected in one pair of markers. These SNP markers are expected to be useful for future quantitative trait loci (QTL) analysis, and to facilitate marker-assisted selection (MAS) in *A. japonicus*.

## 1. Introduction

The sea cucumber *Apostichopus japonicus* (Selenka 1867), naturally distributes along the coasts of China, Japan, Korea and Russia [1]. Due to their nutritional and medicinal value, they have long been exploited as an important fishery resource in East Asian countries. Over the past decade, the aquaculture of *A. japonicus* has become widespread along the coasts of China, due to increasing market demand and over-exploitation of wild sea cucumbers [2]. However, the rapid expansion and intensification of sea cucumber aquaculture has resulted in some severe problems, such as wide-spread disease and stock deterioration, possibly caused by inappropriate broodstock management and inbreeding depression [2]. In order to properly manage broodstock resources and efficiently enhance aquaculture production, control of inbreeding and selection of broodstock with the desired traits, such as rapid growth and disease resistance, are currently necessary for sustainable development of the *A. japonicus* aquaculture. Recently, marker-assisted selection (MAS) has become a valuable tool for selecting individuals with traits of interest [3]. To perform MAS, a large number of genetic markers are usually needed to determine the quantitative trait loci (QTLs) associated with economically important traits.

Single nucleotide polymorphisms (SNPs) have been shown to be the most abundant type of genetic variations in eukaryotic genomes [4], and are currently the marker of choice in a variety of genetic studies, such as high-density genetic linkage mapping and QTL analysis. However, only a limited number of SNP markers have been reported for *A. japonicus* [5–7]. Moreover, molecular markers developed from the expressed sequence tag (EST) databases offer several advantages over anonymous genomic markers, as (i) they can detect variation in the expressed portion of the genome, so that gene tagging could give “perfect” marker-trait associations; (ii) they could alleviate the problem of null alleles which is usually associated with markers developed from the non-transcribed regions; and (iii) they are expected to have greater transferability between species, since transcribed regions are more conserved among closely related species/genera.

Previously, our group has released a large amount of EST data by 454 sequencing of the *A. japonicus* transcriptome [7]. By mining our EST dataset, more than 54,000 putative SNPs have been identified, 200 of which were selected in this study for marker development. SNP validation was performed using 48 *A. japonicus* individuals collected from four natural populations. Genetic parameters of the validated SNP markers were evaluated using 40 *A. japonicus* individuals from a single natural population. These SNP markers will be useful for future QTL analysis in order to facilitate MAS in *A. japonicus*.

## 2. Results and Discussion

**Figure 1:**
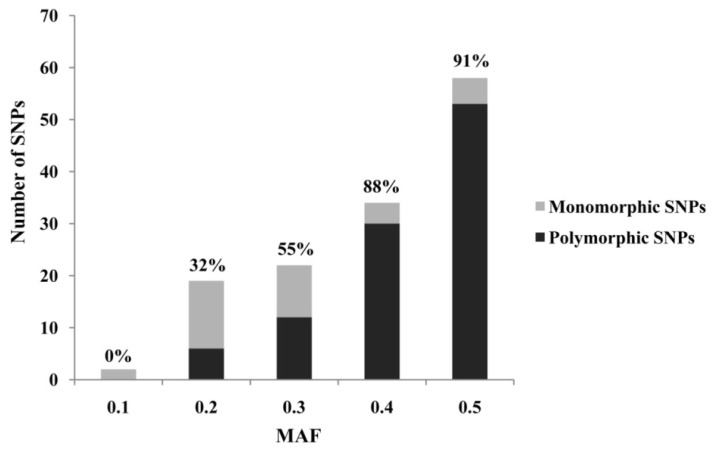
Distribution of SNP minor allele frequency (MAF) for *Apostichopus japonicus*. The number above each bar was the polymorphic rate in respective MAF categories.

Transcriptomic sequences represent an important resource for rapid and cost-effective development of gene-based SNPs. For the high resolution melting (HRM)-based SNP marker development, we designed PCR primers for 200 candidate SNPs (Table 1), which were previously identified from the *A. japonicus* transcriptome generated by 454-FLX sequencing [7]. After PCR amplification, 159 (79.5%) amplified strong bands with expected sizes. The others were discarded without further consideration, as they produced bands larger than expected (possibly caused by introns) or resulted in poor amplification (weak or non-specific amplification). During the initial HRM screen, 63.5% (101) of the 159 successfully amplified loci showed polymorphisms in 48 individuals collected from 4 natural populations, 21.4% (34) generated non-polymorphic curves, and 15.1% (24) displayed unreliable melting curves. In this study, we showed that minor allele frequency (MAF) can serve as an important selection criterion to distinguish true SNPs from sequencing errors when performing SNP mining from 454 sequencing data (Figure 1). For example, most of the validated SNPs usually have a MAF of more than 35%, whereas most non-validated SNPs usually have a MAF of less than 25%. Although our study demonstrated that SNP markers can be efficiently developed from transcriptomic resources, it should be noted that the SNPs obtained may largely represent common genetic variations due to the low coverage of the original transcriptome sequencing, and may suffer from ascertainment bias resulting from simple sample source used in the original transcriptome sequencing.

Genetic parameters of the validated SNP markers were further evaluated using 40 *A. japonicus* individuals from a single natural population. As expected, all 101 SNP loci were polymorphic. The minor allele frequency ranged from 0.035 to 0.489 (Table 2). The *H*o ranged from 0.050 to 0.833, while the *H*e varied from 0.073 to 0.907. Thirteen loci departed significantly (*p* < 0.01) from Hardy–Weinberg equilibrium (HWE) after Bonferroni correction, suggesting that these loci may be under ongoing natural selection. Significant linkage disequilibrium (LD) was detected in one pair of SNP markers (ApjSNP092_CT and ApjSNP098_CT).

As the gene-derived SNPs reside in or are immediately next to protein-coding sequences, they stand a better chance for identifying functional genes that are responsible for complex traits as well as simply inherited traits [8,9]. In our study, 70 SNP markers (Table 2) were developed from the EST sequences showing significant similarity to an entry in the NCBI nr database [10]. Among the annotation information, genes potentially involved in growth or immunity (e.g., epidermal growth factor receptor, Zinc finger protein 62 homolog and heat shock protein 90 kDa beta) were identified. It would be interesting to see whether any of these growth- or immune-related SNPs are highlighted in future QTL mapping of economically important traits, such as high growth rate and disease resistance.

## 3. Experimental Section

### 3.1. Sampling and DNA Extraction

A total of 48 *A. japonicus* individuals used for SNP marker validation were collected from four natural populations (Dalian, Yantai, Qingdao and Wendeng) in China. Genetic parameters of the validated SNP markers were further evaluated using 40 *A. japonicus* individuals from the Rongcheng (Shandong, China) population. Genomic DNA was extracted from the muscles of sea cucumbers by following the protocol developed by Zhan *et al*. [11]. The quantity and integrity of genomic DNA was determined using an Ultrospec™ 2100 pro UV/Visible Spectrophotometer (Amersham Biosciences, Uppsala, Sweden) and gel electrophoresis, respectively.

### 3.2. SNP Discovery and Genotyping

Our group has recently released a large amount of transcriptomic data by 454 sequencing of eight cDNA libraries constructed using more than 200 sea cucumber individuals. Potential SNPs were detected from the assembled contigs using the program GS Reference Mapper (version 2.6, Roche 454 Life Sciences: Branford, CT, USA, 2011) with default parameters (cDNA mode). More than 54,000 putative SNPs were identified from the dataset, 200 of which were selected in this study for marker development with the selection criteria of at least 3× occurrence of the minority allele and at least 6× contigs coverage (number of reads forming the contig). SNP genotyping was performed using a recently developed cost-effective HRM method [12]. For each locus, three non-modified oligonucleotides were used, corresponding to two PCR primers and one probe, primers were designed using Primer3 [13] with the following rules: (1) primer length should be at least 20 bases; (2) product size should not exceed 120 bp in order to decrease the probability of intron interference; (3) the primer Tm should be between 59 °C and 61 °C; (4) the primer GC% should be 40%–60%; and (5) the amplicon contains only one SNP site. Probes were designed using OligoCalc [14] with the following criteria: (1) SNP site locates in the middle of the probe; (2) the length of probe is between 20 and 35 bases; (3) Tm is about 60 °C; (4) the 3′ end of each probe is blocked by two mismatch bases; and (5) no overlap between primes and probe. Each SNP locus was first amplified by an asymmetrical PCR with HRM fluorescent dye in the PCR master mix and then interrogated by an unlabeled probe. The 48 individuals of *A. japonicus* collected from four natural populations were used for SNP marker validation. PCR amplifications were carried out in a 10 μL reaction mixture containing 20 ng of genomic DNA, 1× PCR buffer, 0.2 mM dNTPs, 1.5 mM MgCl_2_, 0.5 U Taq DNA polymerase (Takara, Dalian, China), 0.1 μM forward primer, 0.5 μM reverse primer and 1× LCGreen Plus (Idaho technology inc., Salt Lake City, Utah, USA). The amplification was programmed as: an initial denaturation at 95 °C for 5 min, followed by 55 cycles of 95 °C for 40 s, 60 °C for 40 s and 72 °C for 40 s, finishing with a final elongation at 72 °C for 5 min. The PCR products were checked by gel electrophoresis, and those with correct PCR product sizes were then subjected to probe testing. An aliquot of the appropriate probe was added in each reaction to a final concentration of 5 μM. The PCR product and probe mixture were denatured at 95 °C for 15 min and then slowly cooled to 4 °C. HRM genotyping was immediately performed on a Light Scanner instrument (HR96 model, Idaho technology inc., Salt Lake City, Utah, USA) with continuous melting curve acquisition (10 acquisitions per °C) during a 0.1 °C/s ramp from 40 to 95 °C.

### 3.3. Data Analysis

Data were retrieved and analyzed using the Light Scanner software followed by manual curation of the obtained genotype calls. POPGENE [15] was used to analyze allele frequency, expected (*H*e) and observed (*H*o) heterozygosities, and tests for deviation from Hardy-Weinberg equilibrium (HWE) and linkage disequilibrium (LD).

## 4. Conclusions

In summary, 101 gene-based SNPs were successfully developed from the transcriptome sequences of *A. japonicus*. These developed markers are expected to be useful for future QTL analysis, and to facilitate MAS in *A. japonicus*.

## Figures and Tables

**Table 1:** Results of validation and genotyping of candidate single nucleotide polymorphisms (SNPs).

Categories	Number of SNPs
Total number of tested SNPs	200
Successful PCR	159
Successful genotype calling	135
Polymorphic SNPs	101
Monomorphic SNPs	34
Failed SNPs	65

**Table 2:** Characterization of 101 SNPs for the sea cucumber *Apostichopus japonicus*.

Locus ID	Gene Name	Primers and Probes (5′–3′)	Size (bp)	*H*o	*H*e	MA	MAF	*p*-Value
ApjSNP001_CT	similar to Mech2 protein	F:CCTCAGTCCCAATCACCACT R:ACACTGGCATACACCAGCAA P:CAATGACTTCTCCTTCTCCTACAGCTTCC	98	0.250	0.431	T	0.128	0.239
ApjSNP002_CT	Iron-sulfur cluster assembly 2 homolog	F:TGAATCAGGCAGTTGTGATGA R:GGTCCAGCTAGTCATGCTTTT P:TCAAAGAAATACACTATTTCATCCGATAAAGCAAG	102	0.231	0.485	C	0.397	0.080
ApjSNP003_AC	Protein strawberry notch homolog 2	F:AGCGATTATATCCGATGCAG R:GCTGACCAGGGTAGTTCGAC P:TACAAGAGTAGCCAATCAGAGGCGAGC	108	0.431	0.583	A	0.489	0.482
ApjSNP004_AG	Thiosulfate sulfurtransferase	F:CAGTTGTAACTGCACCTCAGC R:ATGCCTACTTGGATGCCAGA P:ACCACAGGGTGTAGCCAGGTTCGTCAG	70	0.314	0.342	A	0.178	0.578
ApjSNP005_AG	Thiosulfate sulfurtransferase	F:AGGCATCCCTACGGGTATTT R:ACAGGAATGAAGTGGCTTGG P:TCGCTCTAGTCATTCCGCCTCAACG	70	0.374	0.312	A	0.240	0.857
ApjSNP006_CT	Dynein heavy chain 6, axonemal	F:GGGAGGTCTTACGAAGTGGA R:CGAGGAGTTCGGAGTAGCTTT P:ACCCTACCGACTGTCGCAGAGAATG	97	0.271	0.273	C	0.384	0.345
ApjSNP007_AG	Sodium-dependent phosphate transport protein 2B	F:ACCTTGGTGGCAGATATGGA R:TTCAGTGTCCGCAGATTTCTT P:AGTTGAATTAACGGCTCTCGAACCAG	75	0.252	0.432	G	0.287	0.418
ApjSNP008_GT	Testis-specific serine/threonine-protein kinase 1	F:CCACAATTAGCGATGGGTTT R:CAAAGCTCCAGGACTTCTGC P:GTTGTAGTGAACGCATTGGTTTAGGAAGGAAAC	102	0.349	0.488	G	0.410	0.058
ApjSNP009_AC	Disintegrin and metalloproteinase domain-containing	F:CTAAAGGGGATCACCACGAC R:ATAAGCAGGCTTCCCTTTCG P:ACCTTTCGCCGGCCACGCCCT	94	0.208	0.289	A	0.104	0.365
ApjSNP010_GT	Zinc finger protein 62 homolog	F:CCACCAGATGTCTTTGATTCG R:TCACGACCAATACTGCTTGG P:AGATCCGACCCATGCAAGACCAAGGT	106	0.428	0.512	G	0.448	0.552
ApjSNP011_AG	Kelch-like protein 9	F:CAGTCAGCCTAGCCCTACCA R:TCGTTGACCTTTGGTACTGATG P:GTGCAAACCAATCGCAAGTCATTGTCGT	93	0.500	0.498	G	0.458	0.574
ApjSNP012_CT	TATA box-binding protein-associated factor RNA	F:CCTTCACTGGTATGGCATGTT R:TGATCCATGTAGGGAGGCTTT P:GTACATTAACTCTCCACAAGCTCCCTTGTA	88	0.314	0.468	T	0.240	0.045
ApjSNP013_AT	Protocadherin Fat 3	F:TGTTAGCACCTCTATCAAGGATGA R:TTCCATACCTCCTGCCAATC P:GTTCAAGGACACTTGATGGAAAGTGTAATGATT	102	0.500	0.454	A	0.454	1.000
ApjSNP014_GT	Seryl-tRNA synthetase, mitochondrial	F:ATTCGTGTCCAGTTCGCAAT R:GAGATCGGGCGATATAACCA P:TCATATCAATTTGTGCCTCGAGGATCGAC	96	0.271	0.276	G	0.386	0.346
ApjSNP015_CT	Creatine kinase, flagellar	F:TCACAGGCCATCGATCATAC R:CCTTTTCACCAACCTCTCCA P:TCTAAGAGGTGCTGGTGCCCAGTAC	92	0.436	0.502	C	0.446	0.556
ApjSNP016_AG	Fibrinogen-like protein	A F:AATGGCCTCAAGAAAGTGGA R:TCCAGTACCTAGATTTGAAGGACA P:GAATTCATGTGGAGTGAGCATCTTGGAAT	108	0.430	0.583	A	0.483	0.497
ApjSNP017_GT	Abhydrolase domain-containing protein 14B	F:CGGGGTCTACCTCATACAACC R:CCTCCGCCATCTACAGTGTT P:CATATATGGAGCCATTTGCTGTATATTGTAACATG	78	0.293	0.444	T	0.475	0.854
ApjSNP018_AG	Apolipoprotein A–I-binding protein	F:CATAGGTGTCCAGAAATGTTCG R:TGTCCCATGTCTAAAGCATAACTG P:CACAGAGTTCCCATGGGCAGATAGAAG	93	0.073	0.083	G	0.083	1.000
ApjSNP019_AG	*N*-acyl-phosphatidylethanolamine-hydrolyzing	F:CGTGCTCGGTTTTAATGTTG R:CATGGTGAAACCTGGTAGACG P:CCAAGCACAACCAGAACCGAGAAATCCA	91	0.688	0.505	A	0.354	0.498
ApjSNP020_AT	Polypeptide N-acetylgalactosaminyltransferase 11	F:AAAGAGGTATCGACCTTGTCCA R:TGCTCGGACTGTATGTTCATC P:TGGAGGAACTTCCAGAAATCAATGCTGAG	109	0.250	0.256	A	0.328	0.857
ApjSNP021_AG	Hyalin	F:TTCAAGTGGTATCACGAAAACG R:CGTGCTATTGCCTTTGGATT P:GCTGAGGCTTCCAAAAGATGACGATTC	92	0.108	0.333	A	0.290	0.557
ApjSNP022_CG	Transmembrane protein 129	F:TGGAATGCCACTAACACCAA R:TTGACACCACACCACCAATC P:TTGATATGTCTGCTGGGCTATTCTGGTA	80	0.442	0.364	G	0.483	0.381
ApjSNP023_CT	Mediator of RNA polymerase II transcription subunit	F:GCTGATGAGCAATCTTCACACT R:CAAGTTTCAGACGGGACCTG P:GTCTTGATTATCCACGAATCTGTGACATACCA	95	0.146	0.505	T	0.489	0.051
ApjSNP024_AG	AF339450_1 hillari	F:TCCATTGAACGGAGGACTTC R:CAAACATTTCAGCCTTGTGG P:GTCTGGGATGGGATGTAGTCGACACTTA	108	0.419	0.484	A	0.395	0.376
ApjSNP025_AC	Proteasome subunit beta type-5	F:TCCAGATCGCTACGGTCTTC R:ACGACCAGGTAGCTGCAGAG P:TGGTGTATCAAGGAAATTCAAACCCAGCTGT	81	0.250	0.250	A	0.423	0.125
ApjSNP026_AG	Dynactin subunit 5	F:GCCTGTTGCTGTTAACTTTCG R:CTGGCATGTAACTCTATGAAACTC P:GTTAAGTGAAAGTTGACTGCCTCAGTATTGTA	110	0.316	0.365	A	0.461	0.724
ApjSNP027_AG	Apoptosis-inducing factor 2	F:CAGAGAAAGCTGGAGATGATGA R:ATGATTTCAACTGGGCCATC P:GATGATGAACCGCAGAAGGGTTCGAA	88	0.516	0.467	A	0.361	0.324
ApjSNP028_CT	Uncharacterized protein C6orf163	F:ATAGTTGGGTGTGGCTTTGC R:CCGATGCAGTGATGGAAATA P:AAATGTCACCTAACTGTGATTGATCCTCGCC	104	0.209	0.190	C	0.105	0.698
ApjSNP029_AT	F-box/LRR-repeat protein 2	F:CCGTGATCCTAAATGAGGCTA R:CGCTAAGAGTAAGAGAAAGAAGCA P:GCCTAACCATACTGGATTGGCTAGCAGT	98	0.271	0.237	A	0.135	0.762
ApjSNP030_CT	TBC1 domain family member 10B	F:CCGGAGACGTAAAAGCACTC R:TCGTCGTGTCTGGTATCCAC P:AAGTCTGGACAGCTGTTAGCTAAGGGC	91	0.191	0.174	T	0.095	0.754
ApjSNP031_CG	Stejaggregin-A subunit alpha	F:ATCGGTGCTAGACCCAAAGA R:TCCTTCTCTGGTGAATTGATTG P:CATCCCAACGACGGACCGATATGGTA	81	0.150	0.245	G	0.264	0.358
ApjSNP032_AC	Lysine-specific demethylase 6A	F:CGAAGGCAACCAAGTAGGAC R:TGCCACCTCGATCATTTTCT P:CGCTGGTGTTAATAACTTCATAGTCCGTTAC	91	0.138	0.833	C	0.383	0.497
ApjSNP033_AG	ATP synthase subunit beta, mitochondrial	F:GAGTAACAACGGCCCAGAAA R:TACAGTGCCTACACCGGTCA P:GGTCTGACCGCTATTGGGATCAATCTGC	76	0.458	0.467	A	0.232	0.854
ApjSNP034_GT	Ubiquitin carboxyl-terminal hydrolase 8	F:GGCTTGAAGAAACATGGGTAA R:CCAGTAGATTGCATCTTTCCATC P:TCATGTTCACTTCTTTATACCACACGATGACAT	110	0.292	0.314	G	0.035	1.000
ApjSNP035_CT	Uncharacterized protein C7orf26 homolog	F:CGGTGGTGAGGTGTCTACATT R:GGAATAGGCAACTCGAGGAA P:GTCGGTGAAGTACGAAGCCTTCATGAA	76	0.449	0.367	T	0.485	0.498
ApjSNP036_AC	hypothetical protein	F:AAGATGCCAGACAGCAACAA R:CATGACTGCGTCTTCTGCTC P:CAGGAATCTCACAGACGAGAGGGAACT	100	0.545	0.413	C	0.264	0.857
ApjSNP037_AG	DNA replication licensing factor MCM8	F:GGAACCGGAGAGATGACAGA R:CCAGCGTCGTCACCTTTTAC P:AGAGCAAGATCAACAGAATGAGGACAAAGTA	95	0.492	0.502	A	0.458	0.557
ApjSNP038_AG	LRP2-binding protein	F:GATGAAAGTACCTGGGAGGAA R:AGCTGATCATCGGTCCATCT P:GGAGATTGAAGATTGATCCCACTGACAAACTC	83	0.750	0.625	G	0.147	0.381
ApjSNP039_AG	Endoplasmin	F:ATAACGTCGGACGAGCATTC R:AGCAACCACCATCTCTCTGC P:AAGGGTTTGGAGTAAAACAGTCGGATGCCC	76	0.409	0.479	G	0.387	0.051
ApjSNP040_CT	heat shock protein 90 kDa beta	F:CTTTGAAGATATGATGCCCAAG R:TTGTGTTGCTGCAGGGTTT P:ACTCCGATGACCTGCCTCTCAATGTGA	102	0.348	0.291	C	0.174	0.084
ApjSNP041_CT	Titin	F:AGCCATCGAGAATGAGAAGC R:TGATGGTCTGTTCGATCCAC P:GGTCACCGACTACGACAAGATCTCCTGC	82	0.382	0.314	T	0.192	0.091
ApjSNP042_AG	Midasin	F:CAGCCTGGAAGACCCTCAGT R:TTGGACTTCCACCATCAGAA P:AACCAGGCTACGATTTCATGGACCGGT	88	0.800	0.691	G	0.291	0.635
ApjSNP043_CT	Scavenger receptor cysteine-rich type 1 protein M130	F:GGTTCACAACCTCAGGATGAC R:CTTCTGCACACCGCACTTT P:GAAATTACAACCTGCTTTAGTGTCCAGAGATAG	95	0.317	0.505	C	0.476	0.200
ApjSNP044_AC	FK506-binding protein 15	F:TCATACACTCAGGGCATCCA R:GCGTAGGCATATGACGAGAGA P:CAGTTTTGTGAGTGTCTTGACAGTGATAGTGG	90	0.583	0.473	A	0.332	0.149
ApjSNP045_AC	Titin	F:CGTTGAGATCCAAGTCAATGAG R:TGTAGGTGAGTGGTGAACGTG P:TAGAAAGAATGGACAGCGTCCCTGGAGT	105	0.512	0.502	A	0.456	0.897
ApjSNP046_AG	Radial spoke head protein 4 homolog A	F:GGGGAAGATGAGGTAGAAACG R:GCTCATACCGATTCCTGCTT P:ACTCCCAAACCTACCGGAACTTATGTTTTAGA	81	0.113	0.109	G	0.056	0.623
ApjSNP047_CT	Phenylalanyl-tRNA synthetase beta chain	F:TGGCAAATCAATCGGATTCT R:AACGGTTCAATGGTTATCTCTAGG P:CTCAAAGTTTGAGCTTCCAAACCCATGTGGA	102	0.326	0.300	T	0.178	0.653
ApjSNP048_AG	Mitochondrial inner membrane protein	F:CCGATGAGAGGGGTATTCAA R:CCCCCATTCTCGTCTATCAG P:GGGAGAGGTGGGAGAATATCCAGAGATA	98	0.222	0.468	A	0.361	0.002 *
ApjSNP049_CT	Sulfotransferase family cytosolic 1B member 1	F:CCAGGGTAAAGTCAAAGGTCA R:ACTGTAGCCCAGAACGATGC P:TCCTTTCATTTTCCCCTCGTACAAGTCATGT	82	0.524	0.479	T	0.278	0.401
ApjSNP050_CT	RalA-binding protein 1	F:GGTTGAGGAGTTCTTGGGAGT R:CATCAGCATGATCCAACACA P:CTGAATGATTTGCCAACTTGTAACTACACCTTAGA	105	0.250	0.408	C	0.275	0.018
ApjSNP051_GT	Alpha-amylase B	F:TTCGATTCATCTGGTGCTTG R:CTTGACCTTCGCAGGTGTTT P:TGGAGAGAGATCCGTAACATGGTCGAATTGT	107	0.096	0.481	T	0.390	0.005 *
ApjSNP052_GT	Putative vitellogenin receptor	F:CAGTCTGAAAGAACCACTGAAGA R:CGAGTATAGGAGGCTGAAAACG P:GCCCAGAAGATATCGCCTCTCTTCAAATAGG	98	0.411	0.485	G	0.400	0.758
ApjSNP053_CT	UDP-*N*-acetylglucosamine--peptide	F:TCGAAGCTAGATTACTGTGAGCA R:TCTGAAGGAGATGCAGGACA P:TGATTTGGATGGCTCTGGTATAGCACTCA	101	0.071	0.503	T	0.404	0.000 *
ApjSNP054_CT	Kanadaptin	F:CAAGCCGTACATGAAAGCAA R:TGTCCAGGTACGAGTCATCG P:AGAAGAAGAAGAATTGGGCGGACGATCT	88	0.585	0.506	C	0.489	0.307
ApjSNP055_GT	Epidermal growth factor receptor	F:TCACGTTCCACCAGATTTTG R:ATGATGGGGGTAATGGCATA P:TGACCAATAGCATATTCGATGTGATGTCACCA	104	0.253	0.435	G	0.424	0.518
ApjSNP056_CT	hypothetical protein	F:ATGCCACCCTCTTAATCTGG R:CTTGCCTGGGTTTTCCATAC P:TCAGACCGGTGCTTCTGACAGTACATT	107	0.125	0.117	T	0.318	0.442
ApjSNP057_CG	RuvB-like 2	F:CCATAACACCGATGACACCA R:GAAGCTGATAAGATGGAAGTAGCC P:CATTGTCAAGGCAGTCATCTTGTCAGGA	108	0.295	0.388	C	0.258	0.159
ApjSNP058_AG	Eyes absent homolog 1	F:CGTATCCCGTACCACAACCT R:AACCCGTAGGGAACCTGACT P:GGTGTGCAACCAAACGCTGGGTACGG	79	0.400	0.501	G	0.247	0.485
ApjSNP059_CT	WD repeat and FYVE domain-containing protein 3	F:TTCCAGGGATTTGACAGAGG R:TGGCATCTAAAGCTGCTAGTCT P:TCCAGGAGAGATCCTAGGGTGTACTGGG	110	0.530	0.500	C	0.446	0.984
ApjSNP060_AT	similar to LOC398543 protein	F:CCACTACACATCGGTGACCA R:CATCTCCTTCCGATAACACAGTT P:AGATGAAGAATGTATTATTAACGCTGCACACT	110	0.095	0.433	A	0.309	0.008 *
ApjSNP061_CT	Coiled-coil domain-containing protein C6orf97	F:GCTGTTGCCGATGAAACAAT R:CAAATTGAACGAGATGGAGACA P:AGAATATCCTGCCTTGGGATAACGTAAACC	110	0.479	0.447	T	0.329	0.489
ApjSNP062_CT	Uncharacterized gene 48 protein	F:CAGAAGGATAAAGTCCAAGAGACC R:TTCTCCTTTCTGTCCATCCTG P:ACAGGCCTATAGCTACGATCAGGAATCG	86	0.182	0.220	T	0.198	0.809
ApjSNP063_AT	Uncharacterized protein C2orf73 homolog	F:CACATGTGTCACCTCTGGCTA R:ACTGGAACAGCGCCTTTAGA P:CAGCTCAAACCCTCACAACTATGCAAG	73	0.479	0.586	A	0.311	0.252
ApjSNP064_AT	Methionine synthase	F:TCGATACCCTTCACCAAAGAAT R:CGAGGGTCTTGGGAAAGGA P:CCAGGCTTCATCATCAACAGCTTTCTAA	103	0.486	0.495	T	0.432	0.654
ApjSNP065_CT	Tubulin alpha chain	F:CATAGCTTCGGTGGTGGAAC R:GCTTCGATTTCTTGCCGTAG P:GGATTTGCAGCTCTACTTCTTGAACGCG	85	0.061	0.091	T	0.091	1.000
ApjSNP066_AG	TATA element modulatory factor	F:TGGTGCTCAGCTGAATCTGT R:TGGTCTCTTCGTGAGCCTCT P:GAAACAACAAGACAACCTCGAGAGGCTT	86	0.415	0.100	G	0.321	0.007 *
ApjSNP067_AG	TATA element modulatory factor	F:GCAACTGGAGGCAGAGAGAG R:GGCCTGCTCGAGTTTACCT P:AGAGACCAAGGAAGAGCTGGAAGAGAA	79	0.315	0.400	A	0.206	0.486
ApjSNP068_CT	Uncharacterized protein KIAA1704 homolog	F:TGACACCTATGGACCGTCTCT R:GGAGGTAATGGTGGACCAAA P:GGATTCAAAGGTGTCGACAAAGAGTCTGAAC	90	0.412	0.504	C	0.477	0.135
ApjSNP069_CT	WD repeat-containing protein KIAA1875	F:GGGTCTTCCAGCCAATGATA R:ACCACGGCTACGTTTGAGTC P:TACTGGTTGATCGCTCTGGAAGAAACAGGA	103	0.326	0.225	C	0.471	0.390
ApjSNP070_AG	Glycoprotein 3-alpha-l-fucosyltransferase A	F:CCAGGAAGGGGTAGACTTGC R:ATCTCGCCGTTCAAGTTGTT P:CTCAGGAAGTTCTAGAGAGGAAGGATGTC	102	0.528	0.469	G	0.334	0.051
ApjSNP071_AG	N/A	F:CGAAACTATAGTGACCTCTTGGTTA R:CAAGCCCTAGTCTCTTCATTCG P:CAGAATTTCTCTCGAAGTCCTTTGCCAG	104	0.364	0.470	A	0.364	0.189
ApjSNP072_AG	N/A	F:GAGTTAGACCCTCGGCTAGGTA R:GCAAAGAGCCTAGCCTTTAGGT P:TGCATCAGTACTAGCAGCATGGAAAACT	87	0.388	0.412	G	0.333	0.247
ApjSNP073_AG	N/A	F:AAATGTACAGACCCGCATGA R:CTGGAAAAACAGTGTGAACCAA P:TGTAAAATTAATGAGCCGTTCGAACCAAGAG	107	0.225	0.309	A	0.188	0.104
ApjSNP074_AT	N/A	F:GATGGTGAAAATCACGGAGAA R:TTCTATGTCTTGTTGATGCAGAGAC P:CACAATAACCTGGAAATATCAACCTTAGAAGAATTCA	103	0.300	0.404	A	0.275	0.108
ApjSNP075_AT	N/A	F:GACCACGATGACAGCCAGTA R:CTCGCCAAGTCAGGAAAAAG P:AGGATCGTCATTCGGGCACTCTTGG	95	0.630	0.879	T	0.450	0.328
ApjSNP076_CT	N/A	F:AACTCTCGATGGAATGCAAAG R:AACAGACTCGGTCGCATCTC P:GATAGTTCTGACAGCGATTTAGGAGACTAA	108	0.175	0.392	C	0.263	0.001 *
ApjSNP077_CT	N/A	F:AACCATCCTGTAGCGAAACC R:CGGGGACGAGGATATTGTTA P:GTGTTGAATGAAGTCGTTCGCGTAAATGC	103	0.175	0.339	T	0.213	0.004 *
ApjSNP078_GT	N/A	F:GCCAAGCAACATACAGAAGGA R:TAGTTGGGCTGTCTTGCTGA P:TTGCTGCATTAATGTTTAGATGATGATGTGTCT	87	0.563	0.907	T	0.487	0.637
ApjSNP079_AG	N/A	F:TGGGCAGAAGAAAATTTGGA R:GAGTGGCACATGACTTGGTG P:CTGCAATTGGACAACCCCATGCTCAT	99	0.475	0.469	G	0.375	0.084
ApjSNP080_CT	N/A	F:GGGCGCTATCAGACTTTGAC R:GCACCCTCTATTTTAGCTGTTCA P:TCTTGCTAGCTAATGGGAAAGAACGTTAT	110	0.200	0.292	C	0.175	0.062
ApjSNP081_CT	N/A	F:CTGGTTGCAATAGGTTATTTGG R:TGAATACATGCCGTTTCTGA P:GTTGGATTCAGAACACAGACTGCCATTCC	103	0.075	0.073	C	0.038	0.780
ApjSNP082_CT	N/A	F:CAGAAACGGCATGTATTCAAAC R:CCCGACCACAAGGAAAGATA P:AGGGGAGTTTGTGATGACAAATTGTTGCAG	94	0.500	0.404	C	0.275	0.098
ApjSNP083_AC	N/A	F:CACGATGCCCTGTGTGTAAT R:GTCGGCCTCCTGACTAACAG P:GCGCAGCAGAAACGGCGTGGA	108	0.325	0.453	C	0.338	0.073
ApjSNP084_CG	N/A	F:GGGTGGTGCATTTTCTTCAT R:TGGCTTCAGTTACACCATCCT P:ATCCTTGTGGTCGCCTGATCTTGTGTT	75	0.150	0.444	G	0.325	0.000 *
ApjSNP085_AG	N/A	F:CGTCATTCGCTCCAAATACC R:GTCGTAGAGAGACATAACGATAACTGA P:CCATAATGCATAGTGGCTGCAGCATAA	110	0.833	0.896	A	0.487	0.093
ApjSNP086_AG	N/A	F:CGACAATATACTACAAATGCCCTGT R:GATGATGAATGGGTTGTTTGTG P:CAAGGCGAGTTCGTCACACGAAAAGT	83	0.050	0.461	G	0.350	0.000 *
ApjSNP087_AC	N/A	F:CACTCTGGCCTTGCACTCTT R:TGTGAGAACAATAGGTTCACAGGT P:GGGCAAACTGATGTCATGTTCACAGGTATGT	109	0.450	0.353	C	0.225	0.252
ApjSNP088_AG	N/A	F:ATGAAGCATGCGTGAATGAG R:CGATTTCACTGCTGTCATCAA P:AACTGTGGAGATGGTAACATATTCTATGAAGAGAA	83	0.250	0.222	G	0.125	0.256
ApjSNP089_AG	N/A	F:TGGTGAGAAGCATCCACAGA R:GTTGTTTTGAAGGCACTGATGA P:AAGTTCTTAAATGCAGAACTGGGTCAGAACA	93	0.325	0.468	A	0.363	0.051
ApjSNP090_CT	N/A	F:TTGTACCGAGAAAGGGATGTTT R:CCTGAACAACATCTGCCTGA P:AGAGTATATTTCAAACGAAAACGGGAGTAGGGT	110	0.161	0.373	T	0.242	0.002 *
ApjSNP091_CT	N/A	F:TGCGTCATTCTAACCAACCA R:AACACTTATGTAGGCGAGTCTTGA P:CAAAGCGCTTCATTTTCACAGCAACTA	102	0.200	0.380	C	0.250	0.004 *
ApjSNP092_CT	N/A	F:TGACTGGACGTCAGATGTGG R:GTGGGCTTCCAGACACAGAT P:GGTTGCATCAAGGTCCCTGGGTACATACA	81	0.075	0.073	C	0.038	0.780
ApjSNP093_AG	N/A	F:TGAAATGTGGTGTGACTTGC R:TGTGTGACTTCAGCATCTCTGT P:GAATTGTATAATTGGATGCTGTGTGTCACTTAT	80	0.222	0.282	G	0.167	0.227
ApjSNP094_GT	N/A	F:TCTGCTAAGTTGTTGAGAGGATG R:CGAACGGTTGGTATTTGTGA P:TTCTGGTCACTTGCCCCAGGTTCCAC	108	0.171	0.358	T	0.229	0.003 *
ApjSNP095_AG	N/A	F:ATTTGCGGCTCTTCTGTTCA R:TGAAGTGAACTCACCCACGA P:AAACTTGGCAACGAAGACGTCAGCAT	110	0.225	0.367	A	0.238	0.018
ApjSNP096_CT	N/A	F:TCATTCCTGTATTGCTACTACTCTGTG R:TGTGGTATGCCCATCGATTT P:TAAACAATAGTACTTAATGGCATTGAAGACAACAAAC	109	0.333	0.491	C	0.409	0.060
ApjSNP097_CG	N/A	F:CACAGTGATGTGTATGTACGTTCG R:GACCTTCGCTTTGTGCCTAC P:ACACACCGTATATACCGAATCTGGAAATTATCTT	94	0.316	0.337	C	0.211	0.698
ApjSNP098_CT	N/A	F:CTGTGTCAGAGAGGAAGAGTGC R:CGAAAGCTATTTCAAACCCAGT P:GGGTACTATCAAAATTGACTCACAAAGCGAC	107	0.158	0.147	C	0.079	0.512
ApjSNP099_AG	N/A	F:GACCTTCTGCTCTGCCTGAC R:CGGATATCAACAAACCAGAGC P:TCCTCATCTTCGGTGTCTTGCGAAC	97	0.075	0.162	G	0.088	0.080
ApjSNP100_GT	N/A	F:TCCACTGAGCCATCCTGATT R:GAAGAAAAACATGTCCCGATG P:AGTGGCTCCCCCTGGAATGTAATCCTG	103	0.505	0.547	T	0.458	0.279
ApjSNP101_GT	N/A	F:CTGCTGAAGTATGACAACATTAGAGAC R:CTAGTACTTTCTTCTTCAGTAGTTGG P:CTATTGAAAGCTCGATAGGCACATCCTG	109	0.075	0.240	T	0.138	0.000 *

The underlined bases in the probe sequences indicated the positions of the SNPs; *H*_o_, observed heterozygosity; *H_e_*, expected heterozygosity; MA, minor allele; MAF, minor allele frequency; *P*_HWE_, *P* values for Hardy–Weinberg equilibrium (HWE) test;

* , statistically significant after sequential Bonferroni correction.
